# Health-related quality of life in elderly, multimorbid individuals with and without depression and/or mild cognitive impairment using a telemonitoring application

**DOI:** 10.1007/s11136-021-02848-8

**Published:** 2021-05-13

**Authors:** Caroline Lang, Martin Roessler, Jochen Schmitt, Antje Bergmann, Vjera Holthoff-Detto

**Affiliations:** 1 Center for Evidence-Based Healthcare, University Hospital and Faculty of Medicine Carl Gustav Carus, Technische Universität Dresden, Fetscherstraße 74, 01307 Dresden, Germany; 2 Department of General Practice, Medical Clinic III, Faculty of Medicine Carl Gustav Carus, Technische Universität Dresden, Dresden, Germany; 3 Department of Psychiatry, Psychotherapy and Psychosomatics, Alexianer Hospital Hedwigshöhe, St. Hedwig Hospital Berlin, Berlin, Germany; 4 Department of Psychiatry and Psychotherapy, University Hospital Carl Gustav Carus, Technische Universität Dresden, Dresden, Germany

**Keywords:** Telemedicine, Telehealth, Quality of life, Mental disorders, Multimorbidity, Aged

## Abstract

**Purpose:**

Multimorbidity leads to decreasing health-related quality of life (HRQoL). Telemedicine may help to improve HRQoL. The present study was conducted to show (I) differences in HRQoL and changes in HRQoL over time in elderly, multimorbid individuals with and without depression and/or mild cognitive impairment (MCI) using a telemonitoring application (TMA) and (II) associations between engagement with measurements by study participants using a TMA and changes in their HRQoL.

**Methods:**

The present feasibility study was part of a longitudinal intervention study. Recruited general practitioners (GPs) enrolled individuals and assigned them to risk groups according to absence/presence of depression and/or MCI. Depression was assessed using the Geriatric Depression Scale (GDS-15), MCI using the Mini-Mental State Examination (MMSE), and HRQoL using the SF-12. The TMA consisted of tablets, software, and measuring devices. Measured vital data were transferred to a care and case manager for monitoring and possible intervention.

**Results:**

Nine GPs recruited 177 individuals, 97 of whom were included in the HRQoL analysis. Significantly lower physical and mental component summary (PCS/MCS) scores were revealed in study participants with depression, and with both depression and MCI, compared to participants with no mental disorders. PCS scores did not differ between study dates, but MCS scores had significantly increased over time. Participants’ engagement with measurements was significantly associated with an increased MCS score, but not with the PCS score.

**Discussion:**

Depression and/or MCI are negatively associated with the HRQoL of elderly, multimorbid people using a TMA. Engagement of individuals with vital data measurements via a TMA may increase their mental HRQoL. Mentally impaired people should be closely involved as co-designers and experts in development processes of TMAs to benefit from tailored solutions. An individual’s increased mental HRQoL can be a decisive factor in their engagement with a GP treatment regimen and telemonitoring processes.

**Supplementary Information:**

The online version contains supplementary material available at 10.1007/s11136-021-02848-8.

## Introduction

Multimorbidity is a highly prevalent global health problem in elderly individuals [1, 2]. It is defined as the simultaneous occurrence of two or more diseases in the same person [2, 3]. More severe and prolonged chronic diseases can lead to increased physical and mental suffering of elderly people [2]. The resulting consequences can be a significant increase of health care utilization, health care costs, functional impairment, and a poor quality of life [2, 4]. Multimorbidity is associated with a high level of physical suffering and reduced health-related quality of life (HRQoL) [5]. HRQoL, as a multidimensional construct, assesses the subjectively perceived health status of individuals within a holistic approach—not only on the physical dimension, but also regarding psychological and social facets [6]. As a patient-reported outcome (PRO), HRQoL is increasingly assessed to explore the needs and concerns of individuals, to strengthen the relationship between individuals and physicians, and to enhance shared decision making [7, 8], especially in vulnerable individuals with chronic diseases [9]. HRQoL can be measured using generic measuring instruments (patient-reported outcome measures (PROMs)), such as the Short Form-36 (SF-36) or SF-12, with the two main dimensions of physical health component summary (PCS) score and mental health component summary (MCS) score [8]. The use of PROMs in health care aims to ensure targeted and needs-based care, e.g. recognizing and treating deterioration in individuals’ health more quickly. Furthermore it aims to increase HRQoL and satisfaction of individuals by reflecting their needs and concerns [8, 9].

Results of previous studies with elderly people from different countries indicated that an increase in the number of chronic diseases in an individual is associated with a reduced HRQoL [5, 10–12]. In order to regain and maintain HRQoL, telemedicine can beneficially complement the health care routine of elderly, multimorbid individuals [13]. Telemedicine enables healthcare providers to communicate with each other or with the people across spatial and temporal distances with the aim to improve health outcomes by providing information on diagnostics, therapy, rehabilitation, prevention, research and medical decision making in various settings [14, 15]. National and international studies have already shown that telemedicine applications for highly prevalent diseases such as cardiovascular and respiratory diseases as well as cardiometabolic disorders can help to reduce treatment costs and hospitalization rates and to improve individuals’ HRQoL [16–18]. However, existing results are mixed regarding effects of home telemonitoring applications on HRQoL in elderly people with chronic diseases. Results from randomized controlled trials (RCTs) with elderly multimorbid people using a telemonitoring system showed improvements in HRQoL in study participants [19–22], although other telemonitoring RCTs could not achieve congruent results [23–26]. There is also evidence that the effects of telemonitoring on those with mental disorders, such as depression and mild cognitive impairment (MCI), can be promising [27–29]. A longitudinal telemonitoring study in which study participants had to take weekly vital sign measurements detected significant improvements in depression and HRQoL scores [27]. A significant reduction in depressive symptoms in the intervention group compared to the control group could be shown in a telemonitoring RCT with medically frail and homebound individuals [30]. Insignificant changes in HRQoL in study participants with MCI were determined in a telemonitoring study using a quasi-experimental design [31].

HRQoL can be positively influenced by individuals’ engagement with telemonitoring measures. In the present context, participant engagement with measurements via the telemonitoring application (TMA) is defined as the active and exact measurement of vital data via home telemonitoring devices by participants according to the treatment regimen of the responsible general practitioner. An exact measuring behavior of participants will henceforth be referred to as “exact”.

As reported from intervention studies, observation studies, and mixed-methods studies, study participants were highly satisfied with the use of telemedicine applications, and acceptance and adherence were positively rated [32–34]. Adherence of elderly people was reported as being generally higher than in younger individuals [35, 36] and higher adherence rates in male than female study participants were detected [36, 37], with no difference between age groups [37]. Results of systematic reviews showed that telemonitoring for people with MCI has so far focused particularly on technologies that ensure an independent and safe life at home [38, 39] and support their informal and formal caregivers in monitoring affected individuals so they can stay safe at home [40, 41]. Smith et al. reported results from a home telemonitoring study with a small number of elderly people suffering from MCI [42]. The authors concluded that telemonitoring can be beneficial for individuals with MCI. In spite of 10–15% of study participants who failed to respond to the telemonitoring equipment due to their still active and independent status, adherence to the telemonitoring regimen of study participants who were assigned to the intervention group was stable and their mood had improved [42]. Holthe et al. pointed out that the currently existing evidence on the acceptance of assistive technologies for people with MCI is low, which could be due to the difficulty of assessing the measurement results [39].

### Aims of study and research questions

The overall study “Autonomy despite multimorbidity in Saxony through patient empowerment, holistic care for older people with networking of all regional institutions and service providers” (Acronym: ATMoSPHAERE) contributed to the care of elderly, multimorbid people. A significant challenge is the feasible identification of risk groups within the cohort of participants for whom it could be difficult to use telemonitoring measures. These include individuals with MCI or depression—a patient cohort that was excluded or underrepresented in previous studies [43], although the afflicted suffer from the most common diseases of old age. Supported by professionals in the fields of primary care, geriatric medicine, information technology, social services and economics, a regional ecosystem was created that combined both medical and non-medical services on the basis of a medical technology platform. Cross-sectoral networking enabled e.g. the coordination, optimization and rapid adjustment of care plans and the recording of patient needs. Thus, a comprehensive care concept for elderly, multimorbid individuals was developed, which included aspects of prevention, diagnosis, therapy and aftercare/rehabilitation. Its aim was to enable people to lead a largely self-determined life by controlling their own disease-related restrictions. The increase in HRQoL of the study participants (in the following referred to as “participants”), measured with the SF-12, was defined as a secondary outcome in the study [44, 45]. Participants were provided with a TMA, which enabled them to measure vital signs, transfer these data to a care coordination center, and to request home-based assistance and services from regional providers.

#### The research questions of the present analysis were:

- Were there differences in HRQoL and changes in HRQoL over the course of TMA usage between participants with and without depression and/or MCI?
- Was engagement with measurements by participants using the telemonitoring application associated with a change in HRQoL scores?

## Methods

The ATMoSPHAERE study was funded by the German Federal Ministry of Education and Research (grant number 13GW0075F) and was carried out between October 2015 and June 2019. Participant enrollment started in April 2016; the last participant was enrolled in March 2018, with follow-up data being collected until June 2018.

### Study design and study participants

The ATMoSPHAERE study was designed as a longitudinal intervention and feasibility study and was approved by the ethics committee at the Technische Universität Dresden (approval number EK 1012016). The study was performed in the two largest cities in Saxony (Dresden, Leipzig). GP practices from a network of accredited academic teaching practices were included to facilitate recruitment of elderly, multimorbid individuals.

### Recruitment of study participants

*General practitioners* The GP practices were located in the urban area and each belonged to the practice network mentioned above. Study nurses presented the study during network meetings and encouraged interested GPs to participate. Willing GPs were informed in detail about the study and signed a declaration of consent.

*Study participants* Individuals who met inclusion criteria (Table 1) were informed about the study by their GP. Eligibility of individuals was assessed by applying validated instruments measuring cognition (Mini-Mental State Examination, MMSE [46], and clock-drawing test [47]), mobility (Timed “Up & Go” test, TUG) [48] and their independence in everyday life activities (Instrumental Activities of Daily Living assessment) [49]. If eligible individuals decided to participate in the study, they signed a declaration of consent and were informed about the possibility to withdraw their participation at any time.

**Table 1:** Inclusion and exclusion criteria

Inclusion criteria	Exclusion criteria
Age ≥ 65 years	Missing capacity of consent
Multimorbidity (presence of at least two chronic diseases)	Individuals who cannot speak German fluently
Individuals are capable of understanding participant information and consented to the study	Moderate to severe dementia according to ICD-10 or MMSE < 20
Independent operation of television via remote control and/or computer/laptop ≥ 3 times per week	Motoric impairment (TUG: ≥ 30 s in initial measurement, 20–29 s in two repetition measurements)
Unimpaired hearing	Severe psychiatric comorbidities (e.g. schizophrenic psychoses, addictions)
Sufficient motoric and sensory speech ability	Currently participating in a comparable telemonitoring program or participation within the last 12 months
Sufficient eyesight to follow a television program easily	

*ICD* International Classification of Diseases, *MMSE* Mini-Mental State Examination, *TUG* timed “up & go” test

After inclusion, participants were assessed with regard to possible depression using the validated measuring instrument Geriatric Depression Scale (GDS) [50]. According to the results of the MMSE and the GDS, participants were assigned to risk groups (RGs), as shown in Table 2. RGs were defined as such if mental disorders could make it more difficult for the elderly participant to operate the TMA. The study focused on two of the most common mental syndromes in old age: depression and cognitive impairment [51]. The following specific inclusion criteria were applied:

**Table 2:** Specific inclusion criteria—risk groups

Risk group	Specific inclusion criteria
1	No clinically relevant mental disorder: GDS ≤ 5 and MMSE ≥ 27
2	Clinically relevant affective disorder (depression): GDS ≥ 6 and MMSE ≥ 27
3	Clinically relevant mild cognitive impairment: MMSE 26–20 and GDS ≤ 5
4	Clinically relevant affective disorder (depression) and clinically relevant mild cognitive impairment: GDS ≥ 6 and MMSE 26–20

*GDS* Geriatric Depression Scale; *MMSE* Mini-Mental State Examination

### Description of the TMA and the GP treatment regimen

The TMA consisted of the telemonitoring hardware ASUS ZenPad 7.0, Samsung Tab 4, sphygmomanometer, pulse oximeter, body weight scale, and the telemonitoring software Motiva and was provided by the technical project partner Philips Medical Systems GmbH.

Sociodemographic and health data of new participants were collected by study assistants and then transferred to a Care Coordination Centre (CCC). Then a technician from the CCC instructed participants in their homes on the use of the TMA and they had to carry out the first complete measurement and transfer process of their vital data in presence of the technician. After confirmation of having understood the use of the TMA, the care process started. More detailed description of the telemedicine care process for participants has already been described in a previously published paper [52].

According to treatment regimens of participating GPs, participants measured their blood pressure (BP), heart frequency (HF), blood oxygen saturation (SpO_2_), and body weight (BWT) via measuring devices. The frequency of these vital sign measurements was individually determined by each participant’s GP (once weekly, 2–6 times per week, and daily). Vital sign data were transferred from measuring devices to the tablet and to the CCC, which constantly monitored and critically assessed the data for intervention necessity. If any limit values were exceeded, control questionnaires were sent to the participant and a care coordinator contacted the participant by telephone. If intervention was required, the GP was provided with relevant information immediately.

For each participant and vital sign, each complete calendar week may be evaluated with respect to the relationship between actual and recommended frequencies of measurement (see supplement (Table S1)). We classified a participant as exactly adherent in a certain calendar week if both the number of days with measurements and the total number of measurements were in line with GP recommendations. The calendar week was classified as over-measured if the number of days with measurements and/or the total number of measurements was higher than recommended by the GP. In contrast, the calendar week was classified as under-measured if the number of days with measurements and/or the total number of measurements was lower than recommended by the GP.

### Data collection and measures

To examine changes of participants´ HRQoL and symptoms of depression between t_0_ (baseline, installation of TMA), t_1_ (after six months with TMA), and t_2_ (after 12 months with TMA), participants were interviewed by a research scientist via telephone using the validated questionnaires SF-12 [53] and the GDS [50]. The SF-12 was used in the present study to measure HRQoL. The questionnaire covers eight domains of health that are summarized in two summary scores, the PCS and the MCS, both including six items. The scores of the HRQoL domains range from 0 to 100, with higher scores referring to higher quality of life [54]. Several studies have reported the good psychometric properties of the SF-12 in different age groups and specifically in elderly persons [55]. The GDS consists of 15 questions whose sum score differentiates depressive and non-depressive individuals. Research on screening performance in the elderly showed that the use of the GDS allows an appropriate screening of the very old individuals and a reliable detection of a major depression [56].

Data from BP and BWT measurements as recommended by GPs served as the basis for evaluating participant engagement. Due to limited participant and time coverage, two measurement types (heart frequency and blood oxygen saturation) were not included in the analysis.

### Statistical analysis

Baseline characteristics of study participants were analyzed descriptively. Data on measurements/transfer of vital data were also analyzed descriptively regarding frequencies of engagement with measurements, over-measurement, and under-measurement over time (classification according to supplement (Table S1)). Relationships between HRQoL and risk group membership were visualized by box plots. Statistical significance of differences between risk groups regarding HRQoL was tested using Wilcoxon rank-sum tests for unpaired samples. Bivariate associations between participant engagement with blood pressure and body weight measurements, sociodemographic characteristics and PCS / MCS scores at baseline were investigated using the Spearman’s rank correlation test and the Kruskal–Wallis test, respectively. Relationships between HRQoL and participant characteristics were investigated using panel data on the PCS and the MCS gathered at baseline (t_0_), at the six-month follow-up (t_1_), and the 12-month follow-up (t_2_). To explore associations between HRQoL and covariates, linear multilevel regressions were conducted using the PCS and the MCS as dependent variables and participant characteristics as covariates. In addition to the covariates, the right-hand side of these models contains a random intercept at the participant level. This random intercept captures correlation of the participants’ outcomes over time. To explore potential relationships between changes in HRQoL and participants engagement with measurements, linear multilevel regression models for changes in the PCS scores and the MCS scores between t_0_ and t_1_, and t_1_ and t_2_, respectively, were specified. The share of calendar weeks between the PCS and the MCS measurements in which a participant either followed the recommendations of the physician exactly or measured more often than recommended (over-measured) was included as a covariate capturing active study participation. The rationale behind pooling engagement with exact measurement and over-measurement was that both might be expected to be associated with a higher HRQoL relative to a limited study participation as reflected by under-measurement. Hence, exact and over-measurement were combined to generate a single binary indicator capturing active study participation. Sensitivity analyses were conducted by treating the shares of calendar weeks classified as exact or over-measured as separate variables (see supplement (Table S2)). Associations were estimated separately for study participants measuring BP and BWT, respectively. All estimates were adjusted for participant characteristics. Significance levels of 5% and 1% were reported for all regression analyses. Statistical analysis was performed using the statistical software IBM SPSS Statistics for Windows v25.0 (IBM Corp., Armonk, NY) and R v3.6.1.

## Results

### Sample description

#### General practitioners

We recruited a total of nine general practitioners from the network practices. Three general practitioners worked in Dresden, six general practitioners worked in Leipzig.

#### Study participants

**Fig. 1:**
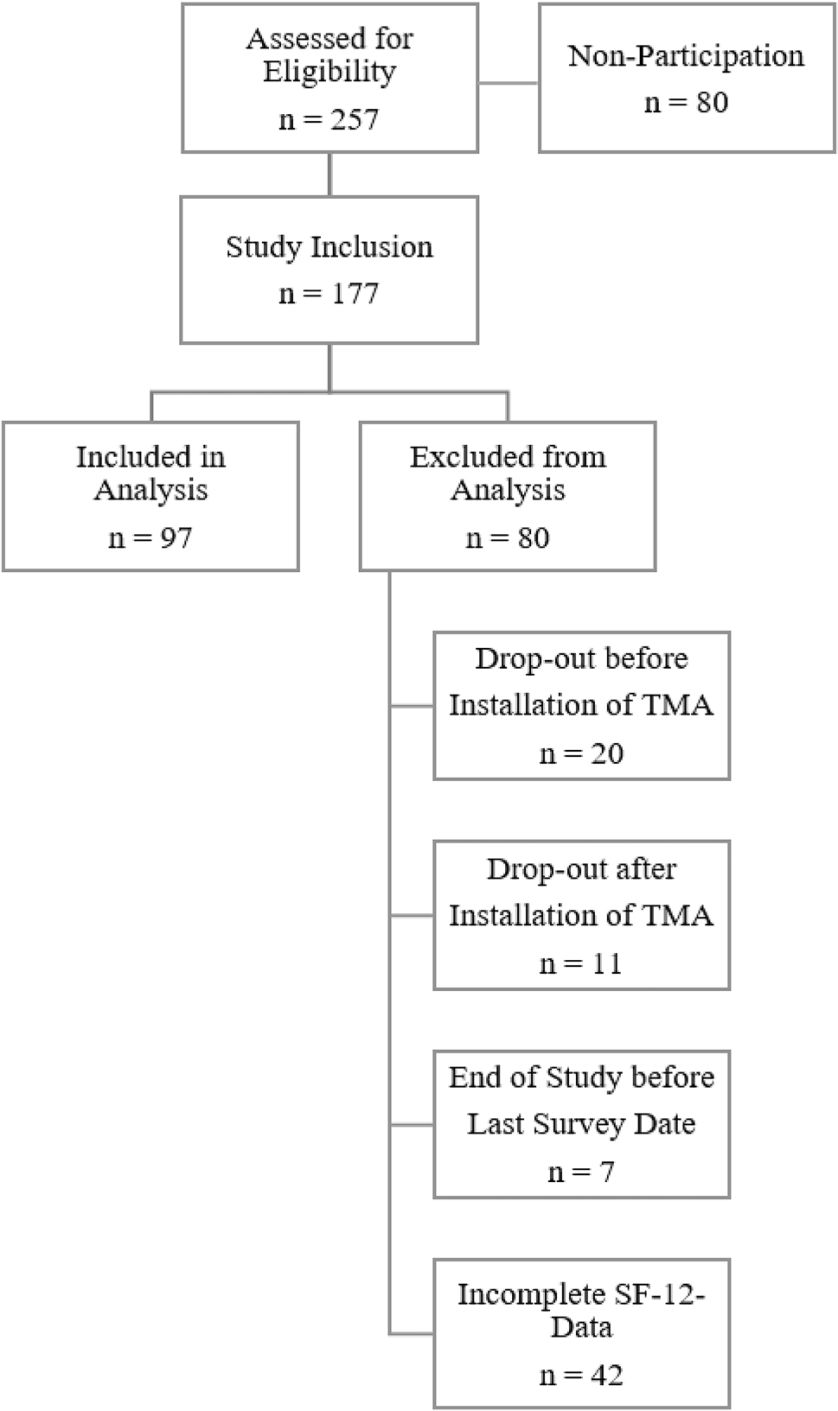
Flowchart of participant recruitment

At both study sites, a total of 257 participants were assessed for eligibility by performing the Geriatric Basic Assessment (Fig. 1). 177 of the 257 screened participants (68.9%) were finally included in the study corresponding to the inclusion criteria. A total of 80 participants screened for study inclusion refused study participation (non-participation, 31.1%). Of the 177 included participants, data from 80 (45.2%) study participants could not be considered for the engagement analysis in advance due to dropout before installation of the TMA (20; 11.3%), dropout after installation of the TMA (11; 6.2%), the end of study before the last survey date (7; 4.0%), or incomplete SF-12 data (42; 23.7%).

More detailed information on the demographic data of the study participants can also be found in previously published papers [52, 57].

In the sample of analyzed study participants, the MCS scores were higher than the PCS scores at baseline (Table 3). According to the German Norming Sample, the mean scores for both sexes at the age of 70 and over is 41.69 (SD 12.1) for the PCS and 52.44 (SD 9.9) for the MCS [54]. Most study participants were required to measure both BP and BWT. Participants were unevenly distributed across RGs, with RG 4 including only four participants. Women accounted for more than 60% of all participants.

**Table 3:** Baseline characteristics of analyzed study participants (*n* = 97)

Participant characteristics	*n*/Median	%/Q1; Q3
MCS, median (Q1; Q3)	55.9	50.2; 59.9
PCS, median (Q1; Q3)	43.5	32.4; 50.4
Vital signs, *n* (%)		
Blood pressure only	30	30.9%
Body weight only	2	2.1%
Both	65	67.0%
Risk group, *n* (%)		
1: Participants free of clinically relevant mental disorders	67	69.1%
2: Participants with depression	10	10.3%
3: Participants with MCI	16	16.5%
4: Participants with depression and MCI	4	4.1%
Age group, *n* (%)		
65–74	21	21.6%
75–85	63	64.9%
86 +	13	13.4%
Sex, *n* (%)		
Male	37	38.1%
Female	60	61.9%
School education, *n* (%)		
Low (< 10 years)	44	45.4%
Medium (10 years)	19	19.6%
High (11–13 years)	34	35.1%
Marital status, *n* (%)		
Alone/widowed	38	39.2%
Married/cohabiting	59	60.8%

*Q1* 1st quartile, *Q3* 3rd quartile

### Differences in HRQoL between risk groups and changes in HRQoL over time

**Fig. 2:**
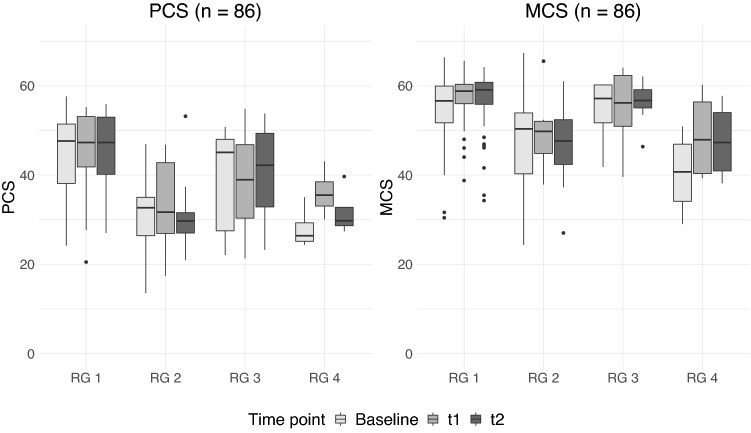
Changes in PCS and MCS scores between risk groups and over time. RG 1 = participants free of clinically relevant mental disorders, RG 2 = participants with depression, RG 3 = participants with MCI, RG 4 = participants with depression and MCI. Scores in the boxplot had not yet been adjusted for other variables (e.g. gender) and considered only those study participants who had complete measurements over all three measurement times

Descriptive analyses of the changes in the physical and mental HRQoL of the participants showed differences between the risk groups (Fig. 2). Statistically significant differences could be determined using Wilcoxon rank-sum tests (see supplement (Table S3)).

The results indicate that the median PCS score of study participants with no depression or MCI (RG 1) remained stable over time and was consistently higher than in study participants with mental disorders (RG 2, RG 3, RG 4). Study participants with MCI only (RG 3) differed only slightly in their median PCS score from RG 1, although this group of study participants showed fluctuations in the median PCS scores over time. Overall, study participants with depression had lower median scores for the PCS than RG 1 and RG 3, but fluctuated only slightly over time. Study participants with both depression and MCI (RG 4) had the lowest median PCS scores at baseline. Their median PCS scores fluctuated over time, but were similar to the median scores of the study participants in RG 2. Statistically significant differences regarding physical HRQoL of participants were found at t_0_ (baseline) between RG 1 and RG 2 (*p* = 0.0013) and between RG 1 and RG 4 (*p* = 0.003). After six months of using the TMA (t_1_), statistically significant differences in RG 1 compared to RG 2 (*p* = 0.001), RG 3 (*p* = 0.024) and RG 4 (*p* = 0.017) were found. At t_2_ there were statistically significant differences in RG 1 compared to RG 2 (*p* < 0.001) and to RG 4 (*p* = 0.005) and between RG 2 and RG 3 (*p* = 0.048).

The MCS score showed a similar picture in the distribution between the risk groups, although study participants’ mental HRQoL scores were higher than the physical HRQoL scores. Participants from RG 1 also showed the highest MCS overall scores as well as the highest MCS scores over time, with the highest median MCS score of all risk groups at time point t_2_. Participants from RG 3 differed only slightly from the median MCS scores of the RG 1 study participants and remained nearly stable over time. Study participants with depression (RG 2) had slightly lower median scores, although their scores also changed only slightly over time. As with regard to the PCS score, the study participants from RG 4 had the lowest median MCS score at baseline; however, this increased over time and became similar to the MCS scores of the participants from RG 2. With regard to the MCS at t_0_, RG 4 showed statistically significant differences to RG 1 (*p* = 0.006) and to RG 3 (*p* = 0.022). In addition, statistically significant differences between RG 1 and RG 2 (*p* < 0.001) after six months of TMA usage (t_1_) were found. At t_2_ there were statistically significant differences between RG 1 and RG 2 (*p* = 0.001), RG 1 and RG 4 (*p* = 0.023), as well as between RG 2 and RG 3 (*p* = 0.015).

The descriptive results withstand an adjustment for other potential covariates of PCS and MCS. Linear mixed effects regression results for the PCS and the MCS levels as dependent variables showed significant differences between risk groups (Table 4). Relative to study participants with no mental disorders (RG 1), study participants with depression (RG 2) and with both depression and MCI (RG 4) had significantly lower PCS and MCS scores. Compared to RG 1, no significant differences were found for participants with only MCI (RG 3). Higher age was associated with lower PCS and MCS scores. Female participants had lower MCS scores than male participants but did not differ significantly from the latter with respect to the PCS scores. The estimation results did not provide evidence for associations between school education and marital status. While the PCS did not differ between study dates (t_0_, t_1_, t_2_), there was evidence for an increase of the MCS over time as reflected by the positive and statistically significant coefficients of the indicator variables for t_1_ and t_2_.

**Table 4:** Coefficient estimates from linear mixed effects regressions for levels of the PCS and the MCS with 95% confidence intervals

Covariate/dependent variable	PCS	MCS
Risk group: RG 1: Participants free of clinically relevant mental disorders (Ref.)	–	–
RG 2: Participants with depression	− 9.39**	− 6.22**
	(− 15.07, − 3.70)	(− 9.99, − 2.53)
RG 3: Participants with MCI	− 4.40	− 0.48
	(− 8.90, 0.11)	(− 3.49, 2.53)
RG 4: Participants with depression and MCI	− 9.83*	− 9.39**
	(− 18.09, − 1.58)	(− 14.82, − 3.95)
Age group: 65–74 (Ref.)	–	–
75–85	− 4.24*	− 1.31
	(− 8.21, − 0.26)	(− 3.94, 1.33)
86 +	− 9.35**	− 4.50*
	(− 15.27, − 3.43)	(− 8.45, − 0.55)
Sex: male (Ref.)	–	–
Female	1.14	− 2.45*
	(− 2.50, 4.78)	(− 4.87, − 0.04)
School education: Low (Ref.)	–	–
Medium	− 0.35	1.71
	(− 4.89, 4.19)	(− 1.29, 4.70)
High	1.02	0.54
	(− 2.66, 4.69)	(− 1.90, 2.98)
Marital status: alone/widowed (Ref.)	–	–
Married/cohabiting	2.56	− 1.53
	(− 1.24, 6.36)	(− 4.06, 0.99)
Time point: baseline—t_0_ (Ref.)	–	–
t_1_	1.29	2.03*
	(− 0.26, 2.83)	(0.40, 3.65)
t_2_	1.07	1.77*
	(− 0.55, 2.68)	(0.08, 3.47)
Constant	45.09**	58.15**
	(39.21, 50.96)	(54.20, 62.11)
Number of observations	280	280
Number of participants	97	97
SD of random intercept	7.04	3.82

*SD* standard deviation

Significance levels: *p ≤ 0.05, **p ≤ 0.01

### Associations between participant engagement with measurements and changes in HRQoL scores

The linear mixed effects regressions for changes in the PCS scores and the MCS scores over time provided evidence for different relationships between the PCS, the MCS, and TMA usage (Table 5). The share of calendar weeks in which a study participant was exact or measured more often than recommended was not associated with changes in the PCS. This finding holds for both BP and BWT. On the contrary, a higher share of exact or over-measured calendar weeks was significantly related to an increase in the MCS over time. Relative to a study participant that measured BP less than recommended in all calendar weeks, a participant that measured as recommended or more often in all calendar weeks was estimated to experience an MCS increase of approximately 3 points. A similar positive and significant association was found for changes in the MCS and BWT measurement with an estimated effect size of 5.8 points. With respect to the other covariates, there was little evidence for significant relationships with changes in the PCS or the MCS.

**Table 5:** Coefficient estimates from linear mixed effects regressions for changes of the PCS and the MCS with 95% confidence intervals

Covariate/dependent variable	Change in PCS		Change in MCS	
Body weight: share of calendar weeks exact or over-measured	− 0.003	–	5.87**	–
	(− 4.79, 4.79)	–	(1.67, 10.06)	–
Blood pressure: share of calendar weeks exact or over-measured	–	1.11	–	2.94*
	–	(− 1.84, 4.05)	–	(0.24, 5.63)
Risk group: RG 1: Participants free of clinically relevant mental disorders (Ref.)	–	–	–	–
RG 2: Participants with depression	− 2.76	− 0.78	− 0.99	− 3.54
	(− 8.32, 2.80)	(− 5.24, 3.68)	(− 5.86, 3.88)	(− 7.63, 0.55)
RG 3: Participants with MCI	0.62	0.39	− 0.79	-0.73
	(− 3.68, 4.92)	(− 3.21, 3.98)	(− 4.55, 2.98)	(− 4.02, 2.57)
RG 4: Participants with depression and MCI	0.2	1.35	1.48	1.23
	(− 6.37, 6.76)	(− 4.85, 7.55)	(− 4.27, 7.24)	(− 4.46, 6.92)
Age group: 65–74 (Ref.)	–	–	–	–
75–85	− 0.48	0.14	1.91	− 0.31
	(− 4.70, 3.75)	(− 3.02, 3.29)	(− 1.79, 5.61)	(− 3.20, 2.59)
86 +	− 1.31	− 0.73	− 2.23	− 3.38
	(− 7.28, 4.67)	(− 5.49, 4.04)	(− 7.47, 3.00)	(− 7.75, 0.99)
Sex: male (Ref.)	–	–	–	–
Female	− 0.06	0.98	1.04	0.52
	(− 3.93, 3.81)	(− 1.85, 3.81)	(− 2.35, 4.43)	(− 2.07, 3.12)
School education: Low (Ref.)	–	–	–	–
Medium	− 1.15	− 1.08	2.39	0.83
	(− 6.17, 3.86)	(− 4.55, 2.38)	(− 2.01, 6.78)	(− 2.35, 4.01)
High	− 1.1	− 0.79	− 0.31	− 1.68
	(− 4.56, 2.36)	(− 3.75, 2.17)	(− 3.34, 2.72)	(− 4.40, 1.03)
Marital status: alone/widowed (Ref.)	–	–	–	–
Married/cohabiting	− 1.03	1.04	− 0.88	− 1.97
	(− 4.95, 2.90)	(− 1.92, 3.99)	(− 4.32, 2.55)	(− 4.68, 0.74)
Constant	2.63	− 0.72	− 1.02	2.16
	(− 3.81, 9.06)	(− 5.39, 3.96)	(− 6.66, 4.61)	(− 2.13, 6.45)
Number of observations	123	180	123	180
Number of participants	67	95	67	95
SD of random intercept	0	0	0	0

*SD* standard deviation

Significance levels: *p ≤ 0.05, **p ≤ 0.01

We found no statistically significant associations between engagement with blood pressure or body weight measurements and risk group assignment, sociodemographic characteristics or HRQoL at baseline (see supplement (Table S4)).

## Discussion

The present study included participants aged 65 years or older with and without depression and/or MCI. We provide important findings for telemedicine research in elderly, multimorbid individuals and changes in their HRQoL over the course of home-based TMA usage while considering an exact measurement behavior of the participants.

Compared to the mean scores of the PCS and the MCS of the German Norming Sample for individuals of both sexes aged 70 years and over (PCS score: 41.69; SD 12.1 / MCS score: 52.44; SD 9.9) [54], our study cohort showed slightly higher mean scores in both the PCS score and the MCS score (PCS score: 42.47; SD 9.9 / MCS score: 55.02; SD 7.7). For MCS, it could be shown that the results of a telephone interview were 2.4 scale points higher than those of a postal survey [54]. Therefore, it cannot be assumed that the physical and mental HRQoL of our participants differ from that of the norming sample. In participants with depression (RG 2), as well as in participants with both depression and MCI (RG 4), our study detected significantly lower PCS and MCS scores compared to participants without those mental comorbidities (RG 1). Previous studies examined the association between multimorbidity and HRQoL and showed that multimorbidity is adversely related more to a reduced physical HRQoL than mental HRQoL [5, 26, 58]. Our findings are in line with these results, showing a negative association between multimorbidity and a reduced physical HRQoL, but we also showed a decreased mental HRQoL in our multimorbid participants with depression as well as with both depression and MCI. However, this did not apply to our participants with no mental disorders (RG 1). There were no differences in the PCS and MCS scores between participants with MCI only (RG 3) and participants with no mental disorders (RG 1). The lack of difference could be due to the fact that participants with MCI were partially supported by spouses or other family members in the operation of the TMA and therefore received no change in their HRQoL, e.g. in relation to a physical or emotional role function. However, especially participants with mental disorders, e.g. those with depression and/or MCI, may benefit from individual and tailored solutions [42] in the care provided by the GP [59], such as an appropriate TMA. In order to keep HRQoL and engagement with measurements constant over time, participants should be closely involved as co-designers and experts in the development process of future telemonitoring projects [60]. Especially participants with MCI can benefit from participating in telemonitoring development processes. Being appreciated for their contribution can strengthen their confidence and empowerment, stabilize cognitive ability and positively shape their illness experience. Pointing out needs which should be especially considered for this target group provides a great added value for implementation of projects with individuals suffering from mental disorders [60, 61].

Further results from the present study indicated that female participants had a lower MCS score than male participants. No gender differences in the PCS scores were found, nor did the participants differ in the PCS and MCS scores in terms of educational level or marital status. Various studies have shown that older women have a lower interest in technology, use less technology and have less confidence in their own abilities when compared to older men when it comes to operating the technology [62–64]. Female participants may not be able to experience greater empowerment or self-efficacy as a result. We examined participants aged 65 and over who had at least two chronic diseases. Our results indicate that higher age was not only associated with a reduced PCS score but also with a reduced MCS score. Reduced physical and mental HRQoL can be attributed to the perceived burden of disease, which increases with age and makes it considerably more difficult for participants to maintain and manage various activities in daily life [10]. It should be noted, however, that the TMA examined was able to support participants in closely monitoring their state of health and giving them a feeling of security, which in our study is expressed in a significantly better MCS score over time. However, this type of TMA can also reach limits, e.g. when elderly people can no longer cope with life on their own due to progressive diseases.

Associations between exact and over-measuring participants and changes in their HRQoL over the study time could be detected: Participants who were exact in their BP and BWT measurements or had even measured more often than recommended by the GP had a significantly better evolution of the MCS than the participants who measured less often. Positive significant associations had been detected between MCS scores and BWT measurements over time. Since the devices for vital data measurement were always available in the participants’ homes, the participants had the opportunity to carry out measurements at their own discretion and thus to control their own state of health. This possibility could have increased the feeling of security and empowerment in the participants [65], which is associated with an increased mental HRQoL. The increase in the MCS scores over time can also be due to the fact that participants may have felt more comfortable with the closely focused care through project participation and more empowered through self-management of their own health care [66, 67]. These benefits were also reported in other home telemonitoring studies [68, 69]. Higher MCS scores in elderly, chronically ill people had also been shown in an RCT that compared telemonitoring and usual care [19]. Another explanation could be that our study participants who showed an increased mental HRQoL over time could better motivate themselves to use the TMA. However, no association between changes in PCS score over time and engagement of participants with measurements was found. This finding is in accordance with former RCTs that investigated home-based TMAs in elderly, chronically ill individuals [24, 25]. Also here, no changes in physical HRQoL over time were observed.

### Limitations and strengths

Our study sample is a non-representative convenience sample. Eligible individuals were easily accessible and recruitable via the participating GPs. Selection bias could exist, as the GPs and the individuals voluntarily participated in the study. We conducted telephone interviews with the participants to assess their HRQoL and possible depression over time using the SF-12 questionnaire and the GDS, respectively. The SF-36 and the GDS have been validated for interviews [54, 70]. A comparison of the survey types with the SF-36 showed that the results for the MCS score in telephone interviews were 2.4 scale points higher than in the postal survey [54]. This may be associated with a tendency towards social desirability among respondents, which could result in a response bias [54]. Participants may have behaved differently because they knew that they were participating in a study and were under observation (Hawthorne effect). By implementing a randomized controlled study design, statements about the effectiveness of the telemonitoring intervention used would have been possible. However, that design was not used in the present study.

We were able to include a vulnerable target group relevant for telemedicine research in our study: multimorbid individuals aged 65 years and over with mental comorbidities. Even if the risk groups examined are not large in number, the results provide important information about the changes in physical and mental HRQoL of these target groups. The results obtained complement the research area of telemedicine applications in elderly people.

### Conclusion

Our feasibility study showed that depression and the coexistence of depression and MCI are negatively associated with HRQoL of elderly, multimorbid people using a TMA. Engagement of individuals with vital data measurements via telemonitoring applications which are in line with the treatment regimen of the GP may increase the mental HRQoL, but not the physical HRQoL of individuals. An increased HRQoL of individuals can be a decisive factor in their constant engagement with telemonitoring measures. In order to examine the effectiveness of future telemonitoring interventions, randomized controlled study designs must be applied. Future research should focus on further determinants that can influence the usability of telemedicine applications for mentally impaired people.

## Supplementary Information

Below is the link to the electronic supplementary material.

Supplementary file1 (DOCX 19 kb)

## Data Availability

The data of the study are not available for public use; data are owned by the ATMoSPHAERE consortium and the authors are not allowed to share them with third parties.

## Code availability

Statistical analysis was performed using the statistical software IBM SPSS Statistics for Windows v25.0 (IBM Corp., Armonk, NY) and R v3.6.1.
