# Supplementary material for: Health-related quality of life in elderly, multimorbid individuals with and without depression and/or mild cognitive impairment using a telemonitoring application
Source: Qual Life Res. 2021 May 13;30(10):2829–41. doi: 10.1007/s11136-021-02848-8 (PMC8481145; doi:10.1007/s11136-021-02848-8)
Supplement: Supplementary file 1 — Supplementary file1 (DOCX 19 kb) [file 11136_2021_2848_MOESM1_ESM.docx]

**Supplement**

*Table S1: Classification of participants’ engagement in specific calendar weeks with respect to vital sign measurement*

| **Number of days with measurements relative to recommended number of days with measurements in calendar week** | **Total number of measurements relative to recommended total number of measurements in calendar week** | **Overall classification of engagement with measurements in calendar week** |
| --- | --- | --- |
| Equality of actual and recommended number of days with measurements | Equality of actual and recommended total number of measurements | Exact |
| Equality of actual and recommended number of days with measurements | Total number of measurements higher than recommended | Over-measured |
| Measurements for fewer days than recommended | Equality of actual and recommended total number of measurements | Under-measured |
| Measurements for fewer days than recommended | Total number of measurements lower than recommended | Under-measured |
| Measurements for fewer days than recommended | Total number of measurements higher than recommended | Under-measured |
| Measurements for more days than recommended | Total number of measurements higher than recommended | Over-measured |

*Impossible combinations have been omitted.*

*Table S2: Results of linear mixed effects regressions for changes in the PCS and MCS scores with 95% confidence intervals using the share of exact and over-measured calendar weeks as separate covariates*

| **Covariate / Dependent variable** | **Change in PCS** | | **Change in MCS** | |
| --- | --- | --- | --- | --- |
| **Body weight:** share of calendar weeks measured exactly | 2.74 |  | 6.96** |  |
|  | (-5.15, 10.63) |  | (0.03, 13.89) |  |
| **Body weight:** share of calendar weeks over-measured | -1.58 |  | 5.23* |  |
|  | (-7.59, 4.42) |  | (-0.04, 10.51) |  |
| **Blood pressure:** share of calendar weeks measured exactly |  | -0.81 |  | 5.49 |
|  |  | (-9.34, 7.72) |  | (-2.33, 13.31) |
| **Blood pressure:** share of calendar weeks over-measured |  | 1.27 |  | 2.71* |
|  |  | (-1.76, 4.30) |  | (-0.06, 5.49) |
| **Risk group:**  RG 1: Participants free of clinically relevant mental disorders (Ref.) |  |  |  |  |
| RG 2: Participants with depression | -2.85 | -0.68 | -1.03 | -3.67* |
|  | (-8.42, 2.72) | (-5.17, 3.81) | (-5.92, 3.86) | (-7.78, 0.44) |
| RG 3: Participants with MCI | 0.6 | 0.46 | -0.79 | -0.83 |
|  | (-3.70, 4.90) | (-3.15, 4.08) | (-4.57, 2.99) | (-4.14, 2.48) |
| RG 4: Participants with depression and MCI | -0.24 | 1.29 | 1.31 | 1.31 |
|  | (-6.90, 6.41) | (-4.93, 7.51) | (-4.54, 7.15) | (-4.39, 7.02) |
| **Age group:** 65–74 (Ref.) |  |  |  |  |
| 75–85 | -0.14 | 0.05 | 2.05 | -0.2 |
|  | (-4.44, 4.16) | (-3.13, 3.23) | (-1.73, 5.83) | (-3.11, 2.72) |
| 86+ | -1 | -0.72 | -2.11 | -3.4 |
|  | (-7.02, 5.02) | (-5.49, 4.06) | (-7.40, 3.18) | (-7.78, 0.98) |
| **Sex:** male (Ref.) |  |  |  |  |
| female | -0.15 | 1.05 | 1.01 | 0.43 |
|  | (-4.03, 3.73) | (-1.80, 3.90) | (-2.40, 4.42) | (-2.19, 3.04) |
| **School education:** low (Ref.) |  |  |  |  |
| medium | -0.81 | -1.16 | 2.52 | 0.93 |
|  | (-5.89, 4.28) | (-4.65, 2.33) | (-1.94, 6.99) | (-2.26, 4.13) |
| high | -1.22 | -0.76 | -0.35 | -1.73 |
|  | (-4.69, 2.25) | (-3.73, 2.21) | (-3.40, 2.70) | (-4.45, 1.00) |
| **Marital status:** alone/widowed (Ref.) |  |  |  |  |
| married/cohabiting | -1.2 | 0.98 | -0.95 | -1.89 |
|  | (-5.15, 2.75) | (-2.00, 3.95) | (-4.42, 2.52) | (-4.62, 0.84) |
| **Constant** | 2.48 | -0.63 | -1.08 | 2.04 |
|  | (-3.97, 8.93) | (-5.33, 4.07) | (-6.74, 4.58) | (-2.27, 6.35) |
| Number of observations | 123 | 180 | 123 | 180 |
| Number of patients | 67 | 95 | 67 | 95 |
| SD of random intercept | 0 | 0 | 0 | 0 |

*SD = standard deviation; significance levels: *p ≤ 0.05, **p ≤ 0.01; MCI = mild cognitive impairment*

*Table S3: Wilcoxon rank-sum tests for differences between risk groups for PCS and MCS*

| **Time point** | **Comparison of risk groups** | | **p-value** |
| --- | --- | --- | --- |
| **PCS** | | | |
| **Baseline (t_0_)** | | | |
|  | RG 1 | RG 2 | 0.001** |
|  | RG 1 | RG 3 | 0.059 |
|  | RG 1 | RG 4 | 0.003** |
|  | RG 2 | RG 3 | 0.224 |
|  | RG 2 | RG 4 | 0.396 |
|  | RG 3 | RG 4 | 0.133 |
| **t_1_** | | | |
|  | RG 1 | RG 2 | 0.001** |
|  | RG 1 | RG 3 | 0.024* |
|  | RG 1 | RG 4 | 0.017* |
|  | RG 2 | RG 3 | 0.254 |
|  | RG 2 | RG 4 | 0.699 |
|  | RG 3 | RG 4 | 0.744 |
| **t_2_** | | | |
|  | RG 1 | RG 2 | 0.001** |
|  | RG 1 | RG 3 | 0.151 |
|  | RG 1 | RG 4 | 0.005** |
|  | RG 2 | RG 3 | 0.048* |
|  | RG 2 | RG 4 | 0.816 |
|  | RG 3 | RG 4 | 0.102 |
| **MCS** | | | |
| **Baseline (t_0_)** | | | |
|  | RG 1 | RG 2 | 0.126 |
|  | RG 1 | RG 3 | 0.829 |
|  | RG 1 | RG 4 | 0.006** |
|  | RG 2 | RG 3 | 0.323 |
|  | RG 2 | RG 4 | 0.247 |
|  | RG 3 | RG 4 | 0.022* |
| **t_1_** | | | |
|  | RG 1 | RG 2 | 0.001** |
|  | RG 1 | RG 3 | 0.512 |
|  | RG 1 | RG 4 | 0.065 |
|  | RG 2 | RG 3 | 0.110 |
|  | RG 2 | RG 4 | 1 |
|  | RG 3 | RG 4 | 0.267 |
| **t_2_** | | | |
|  | RG 1 | RG 2 | 0.001** |
|  | RG 1 | RG 3 | 0.229 |
|  | RG 1 | RG 4 | 0.023* |
|  | RG 2 | RG 3 | 0.015* |
|  | RG 2 | RG 4 | 0.938 |
|  | RG 3 | RG 4 | 0.058 |

*Significance levels: *p ≤ 0.05, **p ≤ 0.01*

*Table S4: Associations between engagement with measurements and sociodemographic characteristics or HRQoL at baseline (t_0_)*

| **Engagement with measurements** | **Sociodemographic characteristics** | **Estimate** | **p-value** | **n** | **Test** |
| --- | --- | --- | --- | --- | --- |
| Blood pressure | PCS | -0.169 | 0.120 | 85 | Spearman’s rank correlation test |
| Blood pressure | MCS | -0.102 | 0.352 | 85 | Spearman’s rank correlation test |
| Blood pressure | Risk group | 1.658 | 0.646 | 85 | Kruskal-Wallis test |
| Blood pressure | Age group | 3.825 | 0.147 | 85 | Kruskal-Wallis test |
| Blood pressure | Sex | 0.303 | 0.581 | 85 | Kruskal-Wallis test |
| Blood pressure | School education | 4.516 | 0.104 | 85 | Kruskal-Wallis test |
| Blood pressure | Marital status | 0.433 | 0.510 | 85 | Kruskal-Wallis test |
| Body weight | PCS | 0.042 | 0.757 | 56 | Spearman’s rank correlation test |
| Body weight | MCS | -0.126 | 0.353 | 56 | Spearman’s rank correlation test |
| Body weight | Risk group | 5.102 | 0.164 | 56 | Kruskal-Wallis test |
| Body weight | Age group | 1.502 | 0.471 | 56 | Kruskal-Wallis test |
| Body weight | Sex | 2.307 | 0.128 | 56 | Kruskal-Wallis test |
| Body weight | School education | 0.454 | 0.796 | 56 | Kruskal-Wallis test |
| Body weight | Marital status | 0.638 | 0.424 | 56 | Kruskal-Wallis test |
